# Dispersive Non-Geminate Recombination in an Amorphous Polymer:Fullerene Blend

**DOI:** 10.1038/srep26832

**Published:** 2016-05-26

**Authors:** Jona Kurpiers, Dieter Neher

**Affiliations:** 1 Institute of Physics and Astronomy, Soft Matter Physics, University of Potsdam, D-14476 Potsdam, Germany

## Abstract

Recombination of free charge is a key process limiting the performance of solar cells. For low mobility materials, such as organic semiconductors, the kinetics of non-geminate recombination (NGR) is strongly linked to the motion of charges. As these materials possess significant disorder, thermalization of photogenerated carriers in the inhomogeneously broadened density of state distribution is an unavoidable process. Despite its general importance, knowledge about the kinetics of NGR in complete organic solar cells is rather limited. We employ time delayed collection field (TDCF) experiments to study the recombination of photogenerated charge in the high-performance polymer:fullerene blend PCDTBT:PCBM. NGR in the bulk of this amorphous blend is shown to be highly dispersive, with a continuous reduction of the recombination coefficient throughout the entire time scale, until all charge carriers have either been extracted or recombined. Rapid, contact-mediated recombination is identified as an additional loss channel, which, if not properly taken into account, would erroneously suggest a pronounced field dependence of charge generation. These findings are in stark contrast to the results of TDCF experiments on photovoltaic devices made from ordered blends, such as P3HT:PCBM, where non-dispersive recombination was proven to dominate the charge carrier dynamics under application relevant conditions.

All efficient organic solar cells consist of at least two semiconducting components with distinctly different electronic structures: a donor with low ionization energy and an acceptor with high electron affinity. When these components are coated from a common solution, upon drying, they form an interpenetrating, partially phase-separated network, comprising donor- and acceptor rich domains. It has been shown that the presence of the acceptor often affects the order and orientation of the polymer chains^1^,^2^. In some particular cases, the acceptor has been shown to completely suppress the crystallization of the polymer component in as-prepared blends^3^,^4^, despite the fact that layers of the pure polymer may exhibit crystalline regions. It is expected that these amorphous regions in the blend exhibit higher energetic and positional disorder than within crystallites.

For disordered materials, excitations (be it neutral excitations such as excitons or charged excitations such as polarons) may undergo thermalization, leading to a time-dependence of their basic properties on different timescales. Typical examples of this are spectral diffusion of excitons^5^,^6^,^7^ or time-dependent charge carrier mobilities^8^,^9^,^10^,^11^,^12^. In accordance with this picture, recent studies on prototypical polymer:fullerene blend devices revealed significant thermalization effects^13^,^14^,^15^,^16^,^17^,^18^. One example is PCDTBT (Poly[*N*-9′-heptadecanyl-2,7-carbazole-alt-5,5-(4′,7′-di-2-thienyl-2′,1′,3′-benzothiadiazole)]) blended with PCBM ([6,^6^]-Phenyl-C71-butyric acid methyl ester). In contrast to neat polymer layers, the PCDTBT:PCBM blend is nearly amorphous^19^,^20^,^21^, independent of device thickness^3^. Transient absorption spectroscopy (TAS) by Howard and coworkers revealed a progressive red-shift with time of the photoinduced absorption feature assigned to polarons on the polymer, which was attributed to the thermalization of holes within the amorphous polymer network^22^. The large shift of the TAS signal (nearly 200 meV) and the long thermalization time (up to 100 ns) was seen as an indication for considerable energetic disorder of the polymer HOMO in the amorphous blend. Interestingly, the combination of TAS with kinetic Monte Carlo (MC) simulations suggested significant thermalization of the electrons in PCDTBT:PCBM as well, with an initial strong relaxation within the first 10 picoseconds, followed by a more gradual decay on the nanosecond to microsecond time scale^22^. As a consequence, hole and electron mobilities were proposed to be time-dependent, with a more pronounced mobility decay of holes due to larger disorder in the HOMO. It was concluded that polarons are extracted and may recombine while still thermalizing^22^,^23^. Note that previous conclusions regarding the initial thermalization of charges in PCDTBT:PCBM blends were based on TAS experiments on freestanding layers, without electrodes and without an external bias applied. Evidence for carrier relaxation at longer time scales (above 1 μs) came from current extraction by linearly increasing voltage (CELIV) while bulk generation time of flight measurements suggested higher order recombination with a possible time dependent recombination coefficient^24^.

Here, we employ the method of time-delayed collection field (TDCF) to follow NGR in an actual device, at charge carrier densities comparable to those found at 1 sun steady state illumination, and biases in the actual working range of a solar cell. We demonstrate that NGR exhibits a continuous slow-down over several orders in time and that most carriers have recombined or are extracted prior to complete thermalization.

## Results

TDCF is a powerful pump-probe technique to study the efficiency of free charge generation and the dynamics of NGR at application-relevant illumination conditions. In TDCF, a short laser pulse creates charges at a set pre-bias, for example, at the maximum power point. After a certain delay, the bias is ramped up to a high reverse bias in order to collect all extractable charge in the layer. If the conditions are chosen such that losses from NGR can be neglected, e.g., by using a short delay and a low fluence, this method directly measures the efficiency of free charge generation. Information on the absolute carrier generation yield can be obtained as a function of electric field (by varying the pre-bias) or photon energy. Studies on a large number of polymer:fullerene, polymer:polymer and small molecule:fullerene blends revealed that this condition is met when choosing a delay time of ca. 10–20 ns and pulse fluences below ~0.2 μJ/cm^2^ 25,26,27,28.

**Figure 1:**
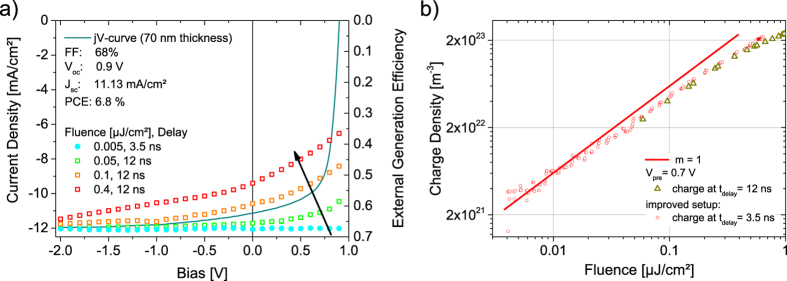
TDCF traces at short delay to probe the field dependence of rapid non-geminate recombination. (**a**) The external generation efficiency of a 70 nm thick PCDTBT:PCBM blend device, measured with TDCF as a function of pre-bias for 3.5 and 12 ns delay time and fluences of 0.005, 0.05, 0.1 and 0.4 μJ/cm^2^. Pulsed illumination was at an excitation wavelength of 532 nm with a laser pulse length of 0.6 ns or 6 ns for 3.5 ns and 12 ns delay, respectively. For comparison, the *JV*-curve of the same device under simulated AM 1.5 G light calibrated to 100 mW/cm^2^ is shown by a solid line. The scaling of the two y-axes was chosen to overlay the low fluence EGE at a reverse bias of −2 V with the current density at the same bias. (**b**) The total collected charge density as a function of laser fluence measured with a pre-bias *V*_pre_ of 0.7 V and a collection bias *V*_coll_ of −3 V.

Surprisingly, when the external generation efficiency (EGE), expressed by the total extracted charge *Q*_tot_ in relation to the number of incident photons, was measured as a function of pre-bias for the PCDTBT:PCBM blend, an apparent field dependence of generation was observed. This is, however, inconsistent with the high fill factor under steady state one sun illumination (AM1.5G, 100 mW/cm^2^), as shown in the comparison between the total charge from TDCF and the *JV*-curve in Fig. 1a. Note that all devices were optimized before the TDCF measurements and exhibited state of the art power conversion efficiencies (PCE), with PCE = 6.8% and 3.9% for an active layer thickness of 70 nm and 220 nm, respectively (see the corresponding *JV*-curves in Figure SI1 in the Supporting Information). Also, these TDCF experiments were performed with a rather short delay time of 12 ns between photo-excitation and the start of the extraction ramp, and a moderate fluence of ca. 0.1 μJ/cm^2^. The apparent field dependence of the EGE in our TDCF experiments, which becomes more pronounced with increased pulse fluence suggests a significant contribution of NGR under the present measurement conditions. A simple way to check for NGR is to measure the total extracted charge *Q*_tot_ as a function of fluence. If NGR is non-linear in free carrier density, this loss will show up in a sublinear dependence of *Q*_tot_ on fluence above a certain threshold value. Figure 1b shows the result of such a control experiment (dark yellow triangles) with a delay time of 12 ns. Interestingly, the charge never scaled linearly with the laser fluence, even for the smallest possible fluence of 0.05 μJ/cm^2^. Based on steady state measurements, the total generated charge should scale linearly with intensity at such low fluences^29^,^30^. This suggests efficient initial NGR in the PCDTBT:PCBM blend at the time scale of a few nanoseconds.

Given that NGR impairs the exact determination of the free charge generation yield even at the smallest fluence and the shortest delay possible with our previous TDCF setup^25^, significant improvements in the sensitivity and time response of the setup were made. Briefly, we enhanced the signal to noise ratio by a factor of 20 by using a new high resolution oscilloscope, combined with a homebuilt, low noise, low offset current amplifier. Furthermore, a laser with a pulse width of 0.6 ns was used. Hereby, the temporal resolution was improved by a factor of four. Also, the high sensitivity of the new setup allows for measurements with much lower fluence. Details can be found in the experimental section. This new setup, made TDCF measurements possible with fluences as low as 5 nJ/cm^2^ with delays of only a few nanoseconds.

With these improvements, full suppression of fast NGR was seen at a very low fluence and at a delay of 3.5 ns. This is shown in Fig. 1b (red circles) where NGR is seen to set in at a fluence of about 0.02 μJ/cm^2^. Field dependent measurements performed under such conditions displayed in Fig. 1a (blue filled circles). These data prove unambiguously that free charge generation in PCDTBT:PCBM blends is indeed field-independent, consistent with other efficient blends^22^,^31^,^32^,^33^. The comparison of Fig. 1a,b shows that great care must be taken to choose a suitable fluence and delay times to exclude NGR, otherwise one may make erroneous conclusions about the FF and *J*_sc_ on the basis of the bias-dependent TDCF experiments.

**Figure 2:**
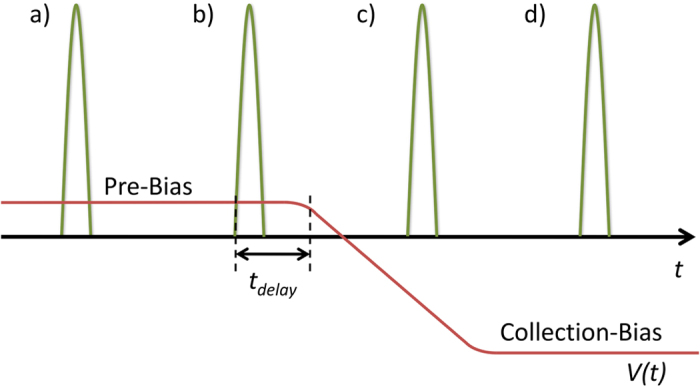
Modified TDCF measurement scheme used to record initial non-geminate recombination losses. TDCF traces are measured at different delays ranging from positive to negative delays. Zero delay *t*_d_ = 0 was defined by the condition that the laser pulse (green line) and the applied extraction voltage ramp (red line) overlay in time at 10% of their final value. (**a**) t_d_ ≫ 0, laser pulse (green) under pre-bias, charges are generated well before collection is initiated and will, therefore, experience significant non-geminate recombination. (**b**) t_d_ > 0, laser pulse under pre-bias but some charges might be lost by fast non-geminate recombination. The example *t*_delay_ shown in the figure (black arrow) is depicted for this case. (**c**) t_d_ < 0, laser pulse under increasing collection-bias, but the final bias not achieved; (**d**) t_d_ ≪ 0, laser pulse under constant collection-bias, geminate and non-geminate recombination is highly reduced.

In order to follow the dynamics of NGR of photo-generated charge in more detail, TDCF experiments were performed with different delays and for a wide range of fluences. Given the fast response time of the improved system, special attention was paid to the fact that due to the sample capacity, the voltage rise across the sample is slower than at the output of the amplifier. Therefore, in addition to classical TDCF experiments, our measurements also included negative delay times, where the collection voltage rise is initiated before the laser pulse hits the sample (see Scheme in Fig. 2).

In the classical TDCF experiment, we start with a large positive delay, for t_d_ ≫ 0 (case ***a***), free charges are generated under *V*_pre_, but undergo significant NGR during the delay before being extracted upon application of the collection bias. When the delay is decreased, (case ***b***, *t*_d_ > 0) generation of free charges still occurs under *V*_pre,_ as in the usual TDCF experiment, but now the voltage ramp is initiated shortly after the laser pulse, and losses by NGR are largely reduced. This is the condition typically chosen to determine the field dependence of charge generation. However, due to the RC-time (*τ*_*RC*_) of the circuit (given by the output resistor of the amplifier, the measurement resistor, the resistivity of the electrodes, the capacity of the sample and of the cables), a certain fraction of the initially generated charges might still recombine non-geminately before the extraction bias is fully applied across the active layer.

While conditions (a) and (b) correspond to the classical TDCF experiment^25^,^26^,^31^,^32^,^33^,^34^, we now also consider cases with a negative delay time, meaning that photo-generation takes place after initiation of the voltage ramp. For a small negative delay *t*_d_ < 0 but |*t*_d_| < *τ*_*RC*_ (case ***c***), the initial bias under which charges are generated and extracted is somewhere between *V*_pre_ and *V*_*coll*_. As the electric field is not yet fully built up in the solar cell, rapid NGR might still take place. At even larger negative delays (situation ***d*** in Fig. 2, *t*_d_ ≪ 0), charge carrier generation takes place at the fully applied collection-bias. Since NGR is strongly suppressed under the full collection bias, all photo-generated charges are extracted to the electrodes. As we showed the EGE to be bias-independent, *Q*_tot_ for *t*_d_ ≪ 0 serves as a reference value with negligible NGR.

**Figure 3:**
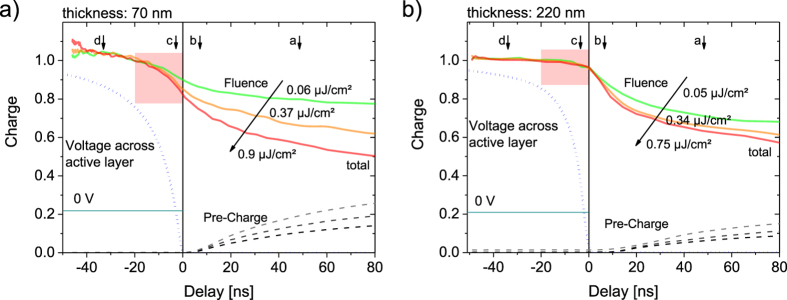
Early time evolution of carrier recombination dynamics in PCDTBT:PCBM devices. The total extracted charge *Q*_tot_ (solid colored lines) and pre-charge *Q*_pre_ (dashed lines) is plotted as a function of delay for different laser fluences including negative delays as described by Fig. 2 for 70 nm (**a**) and 220 nm (**b**) active layer thickness. Data have been normalized to yield the same total charge at a delay time of −35 ns. Also shown is the build-up of the electric field across the device as estimated by integrating the capacitive transient in the dark over time (dotted blue line). The situation where short circuit condition (0 V across the active layer) is reached is indicated by a dark green line. Recombination already sets in during the rise of the extraction field for the 70 nm thick sample, but not for a 220 nm active layer thickness. For the thick (thin) device, an active area of 1 mm^2^ (0.5 mm^2^) was chosen to closely match the RC-time of both devices. The experimental conditions where the same as in Fig. 1b, with *V*_pre_ chosen to be close to the maximum power point at 1 sun illumination.

Results of these measurements are shown in Fig. 3a for a layer thickness of 70 nm. As a consequence of the high capacity of this device the RC-time is ca. 12.5 ns and the electric field takes up to 20 ns in order to be fully applied across thin devices. On the other hand, with an initial pre-bias of 0.7 V and a collection bias of −3 V, short circuit condition (0 V across the sample) is attained already after ~2 ns (e.g. Fig. 3, blue dotted and green solid line). According to the *JV*-curves in Figure S1, short circuit, or applying any negative bias is sufficient to suppress most NGR losses under steady illumination conditions.

In such thin devices, a significant reduction of *Q*_tot_ is observed even at short negative delay, at a condition denoted as case ***c***in Figs 2 and 3a (red rectangle). As free charge generation is independent of field, this loss must be entirely due to NGR. Accordingly, we observed greater losses with increasing fluence. Our data therefore prove that, for an optimized layer thickness of the PCDTBT:PCBM blend, there is efficient NGR at fast time scales. Notably, strong NGR losses already occur during the build-up of the electric field, and can be only suppressed when applying a sufficiently large reverse bias (the reason for this will be discussed later). This rapid (bias-dependent) NGR is the reason for the apparent field dependence of the extracted charge carriers in Fig. 1 for fluences above 5 nJ/cm^2^.

When discussing possible reasons for this early-time NGR, it is worth noting that no such effect was seen in the TAS experiments by Howard *et al.*^22^ despite using higher fluences. They showed that NGR sets in no faster than 10 ns at a fluence of 7 μJ/cm^2^. The two experiments, TAS and TDCF, differ mainly in the fact that while TAS is performed on freestanding thin films, TDCF is measured on the full device structure, with the active layer sandwiched between two electrodes. We, therefore, propose that the rapid NGR seen here is mediated by the contacts.

In agreement with this interpretation, the recombination dynamics are considerably altered when increasing the layer thickness to 220 nm. Note that this sample had a larger active area (ca. 1 mm^2^), meaning that the capacity (and with that the RC rise time) was only slightly smaller than for the 70 nm sample (with an active area of 0.5 mm^2^). With an active layer now three times thicker, the initial NGR loss at *t*_d_ < 0 becomes very much diminished and *Q*_tot_, recorded as a function of *V*_pre_ for a short delay time of 3.5 ns is now essentially independent of fluence (see Figure SI2). An intermediate situation is encountered for an active layer thickness of 150 nm. Note that the thickest device exhibited a weak bias-dependence for all fluences which we assign to a weak field dependence of free charge generation, possibly originating from a slightly different morphology in the thick device^20^.

The above data provide consistent evidence for an initial (fast) NGR loss, which is most pronounced for the thinnest device and is most prominent at biases near open circuit. While the first observation hints at the role of electrodes (or the electrode near regions) in assisting the rapid initial recombination, the exact mechanism still remains to be resolved. One idea is, that hot holes and electrons are generated in the bulk of the active layer, and move within the same direction in order recombine in the surface near region. Evidently, upon reducing the applied bias, an internal electric field is created which redirects one type of charge carriers, thereby suppressing the rapid NGR pathway as described above. In addition, increasing the internal field will speed up extraction of the photogenerated carriers, thereby reducing the probability for NGR. A comparison of the thin and thick layer of 3a and 3b shows that the charges can be more easily extracted from the thinner device i.e. the *Q*_pre_ increases more rapidly with longer delays in the thin 70 nm device.

**Figure 4:**
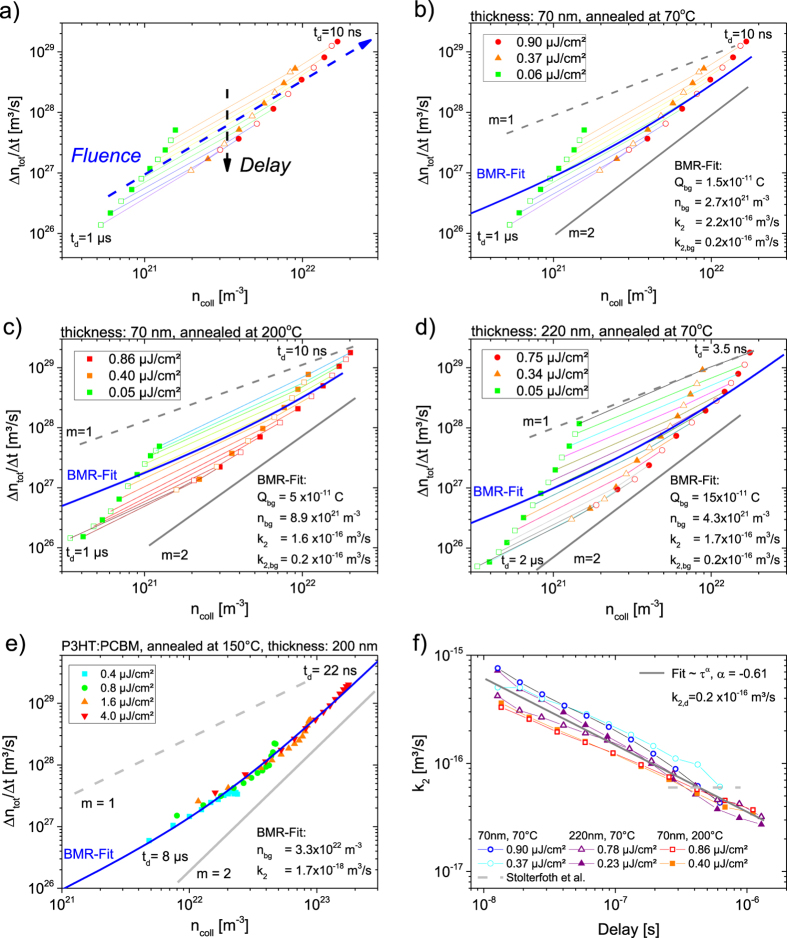
Dispersive Recombination Dynamics in PCDTBT:PCBM. Figures (**a–e**) are differential decay plots showing the charge loss per time unit versus remaining charge in the device for three different fluences. The Pre-Bias was 0.7 V in all cases. The delay increases from the top (10 ns) to the bottom (1 μs), the fluence increases from left to right. Charge losses for 70 nm devices annealed at (**b**) 70 °C and (**c**) 200 °C, and a device with a (**d**) 220 nm thick active layer annealed at 70 °C For comparison, the solid gray line represents a recombination order of 2, the dashed line an order of 1. (**e**) 200 nm P3HT:PCBM device for four different fluences, data depicted from ref. 37. (**f**) The time dependent recombination coefficient in PCDTBT:PCBM nicely follows a power law (grey), which is explained by thermalization of photogenerated carriers in an inhomogeneously broadened DOS. The dashed gray line denotes the recombination coefficient determined from previously measured values of the carrier mobilities and the Langevin reduction factor^40^.

We now turn to the TDCF dynamics at *t*_d_ > 0 (the classical TDCF measurement scheme), which we assign later to bulk recombination. Data showing the dependence of *Q*_tot_ and *Q*_pre_ for a delay time of up to 2000 ns are shown in the SI (Figure SI3). These traces have been normalized the same way as in Fig. 3 for easy comparison. The much more pronounced initial recombination loss in the thin sample is, therefore, clearly revealed by the dependence of *Q*_tot_ on fluence at even short delays. As attempts to fit these traces with the established iterative approach^25^,^33^, using a constant, time independent recombination coefficient, turned out to be unsuccessful, We therefore analyzed these data with a new approach as outlined in refs 35 and 36. In short, the incremental change of the total extracted charge density per time increment, Δ*n*_*tot*_/Δ*t*, with *n*_*tot*_ = *Q*_*tot*_(*eAd*), is plotted versus *n*_*coll*_ = *Q*_*coll*_(*eAd*) for different delay times and fluences. As Δ*n*_*tot*_(*t*) is the loss due to NGR and *n*_*coll*_(*t*) is the carrier density in the device at time *t*,


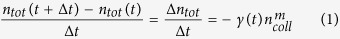


where *m* is the order of recombination and γ(t), the (time-dependent) recombination coefficient. According to equation (1), the slope of Δ*n*_tot_(*t*)/Δ*t* versus *n*_coll_(*t*), plotted for the same delay but with increasing fluence in a log-log fashion, yields the order of NGR at the given delay. On the other hand, any time dependence of the NGR coefficient will show up as a gradual change of Δ*n*_tot_/Δ*t* for a fixed *n*_coll_(*t*) with increasing delay. This general analysis scheme is detailed in Fig. 4a for the 70 nm thick blend, where the fluence increases from the left to the right side and the delay increases from the top to the bottom of the graph. All differential plots were derived from experimental traces of *Q*_tot_ and *Q*_coll_ versus delay time. Scatter was reduced by fitting the original traces with at least three stretched-exponential functions as shown in Figure SI3. The resulting differential losses were then binned logarithmically to reduce the number of data points in the differential decay plots.

For all PCDTBT:PCBM blends studied here (which also includes a 70 nm blend annealed at 200 °C), the incremental loss at a given *n*_coll_(*t*) slows down considerably with increasing delay. This points to the NGR coefficient being strongly time dependent. The situation is very different for a well-crystallized thermally annealed P3HT:PCBM sample (see Fig. 4e, data from^37^), where incremental recombination data for a wide range of fluences and delays lay on the same line, implying a time-independent recombination mechanism at all times studied here. This is in accordance with insignificant spectral relaxation in TAS experiments on P3HT:PCBM blends^38^. Also, recent measurements with transient electric field induced second harmonic generation (TRFISH) experiments on P3HT:PCBM revealed the carrier mobility (averaged over electrons and holes) to decay rapidly at the picosecond range, reaching steady state values within the first few nanoseconds^15^. We, therefore, attribute the pronounced slow-down of recombination in the PCDTBT:PCBM sample to the amorphous nature of this blend.

Note that a quick look at the differential recombination plots in Fig. 4 would suggest that slow-down of recombination proceeds over a longer time scale in the 220 nm sample, compared to the thinner devices. Two underlying causes lead to this outcome. First, there is nearly no initial NGR loss for this thicker device, meaning that the differential plot captures nearly all recombination events. Secondly, 6 times the volume, the 220 nm devices contains a larger amount of charge than the thinner device, allowing us to follow the recombination dynamics with high precision to longer delay times. In fact, as outlined in more detail below, the kinetics of bulk recombination is nearly the same for all three devices.

Before addressing this point in more detail, we turn to the order of recombination. As a general conclusion, we find that the apparent recombination order (the slope of the equitemporal lines in Fig. 4) always falls in the range between 1 and 2 for all samples. This is because recombination of the photo-generated charge also occurs with the background charge in the active layer (due to dark injection or unintentional doping). In previous analysis of TDCF data, bimolecular recombination with background charge was accounted for via


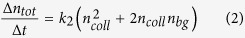


where *n*_bg_ is the density of background charge^25^,^32^,^33^,^37^,^39^. The situation is more complicated for the present PCDTBT:PCBM blend, because background charge is (fully) thermalized while most photogenerated charge is not, meaning background and photogenerated charges may have different k_2_ values. To cope with this problem we assume that the dynamics of dispersive recombination are dominated by the thermalization of a single charge carrier, only. This assumption is rationalized by the fact that transient photocurrent measurements as well as Monte Carlo simulations of TAS data consistently revealed much higher electron than hole mobilities^22^,^40^ across the relevant timescales. We, therefore, expect the motion of electrons to dominate the rate of NGR. In agreement with this interpretation we find that blends annealed at 70 °C and 200 °C exhibit almost the same recombination dynamics, despite the fact that annealing significantly broadens the DOS of the HOMO of PCDTBT and negatively affects charge transport^20^. However, in calculating the recombination order and constant as described below, no inherent assumptions are made about which carrier’s mobility is primarily responsible for recombination.

With this in mind, the differential decay plots were analyzed with


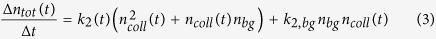


where the recombination of photogenerated electrons is described by a time dependent *k*_2_(t), irrespective of whether the recombination partner is a photogenerated hole or a background charge. Following the same line of arguments, the same time-independent recombination coefficient *k*_2,bg_ is used for the recombination of background electrons with either photogenerated or background holes. To get a reasonable estimate of *k*_2,bg_, NGR in the 220 nm device was analyzed with Equation (2) for the longest delay (2 μs), yielding *k*_2,bg_ ≅ 2 × 10^−17^ m^3^/s. The density of background charge (Figure SI4) was determined independently by applying bias assisted charge collection (BACE), using the very same bias conditions as in our TDCF measurements but with the sample kept in the dark^39^. These values are listed in the corresponding differential decay plots. With this information at hand, the differential plots were fit with Equation (3), with *k*_2_(t) as the only variable. Excellent agreement to the data is obtained, as demonstrated with the solid blue lines in Fig. 4(b–d). We, therefore, conclude that NGR in PCDTBT:PCBM is strictly bimolecular in nature, but with a time-dependent bimolecular recombination coefficient. The only exception is the early time recombination of the 220 nm thick devices, which follows a first order process. We attribute this to an initial free carrier loss by trapping, in line with the interpretation of transient photocurrent experiments by D. Moses and coworkers^41^.

The BMR coefficient *k*_2_(*t*) extracted with Equation (3) from the TDCF measurements is plotted as a function of delay time in Fig. 4f. The plot summarizes data from two fluences for all three devices. It reveals a common power law-type time dependence of *k*_2_(*t*), which we assign to dispersive free carrier recombination in the amorphous bulk of the active layer.

## Discussion

Our studies, performed on complete PCDTBT:PCBM devices, reveal a significant time dependence of the recombination coefficient, compared to well-ordered samples as studied previously before with TDCF^25^,^32^,^33^,^34^,^37^,^39^. Previous experimental evidence for time dependent NGR dynamics came mostly from pump-probe measurements on freestanding blend layers (without electrodes), e.g. by TAS, transient microwave conductivity (TRMC) or transient terahertz photoconductivity^16^,^42^,^43^,^44^,^45^,^46^,^47^, or on complete devices with transient photocurrent techniques such as CELIV^48^,^49^. Though TAS and TRMC exhibit sufficient time resolution to capture the entire NGR dynamics, their limited sensitivity usually requires higher carrier densities. Also, such contactless methods are (by definition) insensitive to the effect of the contacts or an electric field on the recombination dynamics. CELIV, on the other hand, is capable to work with application-relevant carrier concentrations, but the time resolution of this method is quite poor (above 1 μs). With our improved TDCF setup, we come into the position to follow, for the first time, time-dependent NGR for a complete high performance polymer-based device over a wide range in fluence and time, covering the nanosecond to microsecond time period at which charge extraction occurs under relevant operation conditions.

Most importantly, for the thin 70 nm device, we find a fast nonlinear recombination loss, which is only fully suppressed when a significantly large reverse bias is applied. As a consequence, TDCF experiments conducted under “normal” conditions (ca. 10 ns delay time, fluence above 0.1 μJ/cm^2^) yield an apparent field dependence of generation. It is only when the delay time and the fluence is further reduced that the true effect of bias on the efficiency of free carrier generation is measured. Surprisingly, despite the rather amorphous nature of the blend, free carrier formation is found to be nearly independent of field.

**Figure 5:**
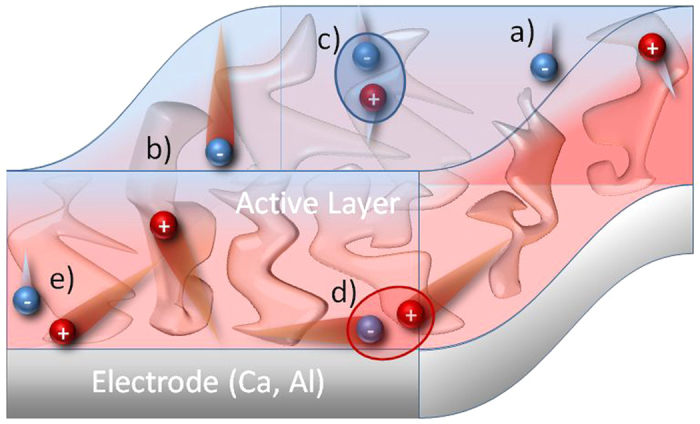
Fast and slow non-geminate recombination in PCDTBT blended with PCBM. The scheme sketches the blend morphology with one example electrode; (**a**) “cold” and slow charge carriers, (**b**) “hot” and fast charge carriers, (**c**) slow non-geminate recombination path between cold charge carriers, (**d**) charges travelling through the device in the same direction, (**e**) fast non-geminate recombination between hot charge carriers near the electrode.

By studying active layers of different thickness, we arrive at the conclusion that the initial fast NGR is mediated by the contacts (or surface-near regions). This situation is outlined in Fig. 5. After the creation of excitons by a short light pulse, hot electrons and holes are generated in the bulk, some of which diffuse in the same direction towards the contacts where they recombine rapidly (Fig. 5d,e). In accordance with this, the early loss is highly field-dependent, as a reverse bias will redirect one type of charge carrier, and prevent it from recombining with the other carrier.

A slight dependence of the fast recombination process on excitation wavelength, especially at open circuit condition, nicely supports that the initial fast recombination takes place (in part) in the vicinity of the electrodes (see Figure SI5). The data show that the fast recombination loss becomes more dominant when the excitation wavelength is changed from the absorption maximum (where the generation profile maximum is in the middle of the active layer) to the near infrared (with the excitation profile moving closer to the PEDOT:PSS anode). Increasing the active layer thickness reduces the probability that photo-generated carriers move close to the contact via diffusion, consistent with our finding that thicker devices don’t display such an initial decay.

At a longer time scale, the recombination dynamics are governed by a second order process, with a continuous reduction of the recombination coefficient following a power-law-type behavior according to 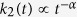. We attribute this to NGR in the bulk, as the kinetics of this process are independent of layer thickness (see Fig. 4f). Such power-law-type time dependence of *k*_2_ is consistent to the results from Monte-Carlo-simulations on bimolecular recombination in disordered materials in the presence of Gaussian or exponential energetic disorder^13^,^50^. These particular decay kinetics were attributed to the continuous thermalization of carriers in the inhomogeneously broadened DOS, which progressively slows down carrier motion. In our PCDTBT:PCBM samples, a power law-type decay is shown to proceed throughout the entire time scale studied here, until all charge carriers have either been extracted or recombined, meaning that the carrier distribution in PCDTBT:PCBM never reaches thermal equilibrium. Importantly, Fig. 4f reveals the decay dynamics are not affected by the initial carrier concentration. This implies that under all conditions employed here, including the presence of (dark-injected) background charge, a sufficient number of unoccupied states are always available for the thermalization of photogenerated charge in the tail of the DOS.

The dispersive recombination reported here implies a significant slow-down of carrier motion in the time range considered, in agreement to the TAS and simulation data published in refs 18 and 22. Figure SI6 compares the Langevin recombination coefficient 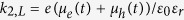 calculated from the time dependent mobilities in ref. 18 to the time dependent BMR coefficients measured here. This comparison suggests that k_2_ is only slightly reduced compared to Langevin recombination at early times, while it relaxes more quickly than would be expected from hole and electron mobilities alone. As a consequence, 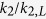 falls below 0.1 on longer timescales; consistent with recently published Langevin reduction factors in PCDTBT:PCBM^40^,^51^.

Our studies show that non-geminate recombination in PCDTBT:PCBM is bimolecular at all times and intensity studied here. This is in accordance with the findings from a large number of experiments on other organic solar cells, with various methods including transient and steady state techniques^52^. This, however, does not imply that photocurrent losses under steady state illumination exhibit a similar nonlinear dependence on intensity in the application-relevant bias range. Near short circuit conditions and at reverse bias, extraction of photogenerated charge is rapid and losses due to non-geminate recombination are weak. Therefore, the photocurrent is nearly equal to the photogeneration current, which is linear in intensity. On the other hand, as the bias approaches V_oc_, the density of dark-injected charge carriers increases substantially, and the non-geminate photocurrent loss is dominated by the recombination of photogenerated charge with dark charge, resulting in a pseudo-linear recombination loss^53^. For PCDTBT:PCBM, as free charge generation is field-independent, the only process which determines the field dependence of the solar cell current under illumination is indeed non-geminate recombination, which is shown here to be dispersive and mediated by the contacts.

## Methods

### Sample Preparation

Devices were fabricated on pre-structured ITO coated glass substrates (Lumtec), which were cleaned in acetone, detergent, DI-water, isopropanol and dried under nitrogen flow. Afterwards, the ITO was plasma cleaned and subsequently a 30 nm layer of PEDOT (Clevios PVP AI 4083) was spin cast on ITO. Annealing of the PEDOT:PSS was performed under inert atmosphere at 180 °C for 10 min. PCDTBT (Mn = 65–85 kDa) was provided by Solaris Chem. Inc.^54^. PCBM (phenyl-C 71 -butyric acid methyl ester) (Solenne BV) was mixed with the donor polymer in a 1:3 wt. ratio (donor:acceptor). 70, 150 and 220 nm thick films of PCDTBT:fullerene blends were spun from solution (35 mg/mL) in dichlorobenzene. On top, a 5 nm TiO_2_ layer was spun from a TiOx precursor solution 1:70 vol. in methanol^55^. Devices were finalized with a Ca/Al electrode.

### Solar cell characteristics

The solar cell characteristics were measured with a Newport Oriel Sol2A simulator calibrated to 100 mW/cm^2^ and a Keithley 2400 source/measure device. The samples were held at 25 °C during measurement. The sun simulator was calibrated with a KG3 filtered silicon reference cell calibrated at Fraunhofer ISE.

### Time Delayed Collection Field

The measurement scheme for the setup used before was described in ref. 33. For the new setup the excitation was done with a diode-pumped, Q-switched Nd:YAG laser (AOT1 picolo, 1 kHz rep-rate, 600 ps pulse duration, 250 ps Jitter). The current through the device was measured via a grounded 10 Ω resistor in series with the sample and recorded with a new homebuilt differential current probe and an Agilent DSO9104H oscilloscope. In order to improve the time resolution of the setup further, additional changes were needed: First, an Agilent 81150 A pulse generator with a fast slew rate of 2.5 ns was used to apply the pre- and collection bias to a homebuilt amplifier: this was directly connected to the sample, such that the cable length is only 5 mm. Second, to reduce the effect of the laser jitter on the measurement, the pulse generator was triggered via a fast photodiode (EOT ET 2030TTL). Also, to compensate for the internal latency of the pulse generator, the laser pulse was delayed and homogeneously scattered in an 85 m long silica fiber (LEONI), which, in combination with an achromatic doublet, resulted in a nearly flat-top beam profile. The fluence was determined with a CCD-camera in combination with a calibrated photodiode sensor (Ophir) and a laser-cut high-precision shadow mask to define the illuminated area. Charge carrier dynamics were measured starting with a negative delay between 50 ns and up to 2 μs with 150 different delays. Each transient was averaged 250 times and the experiment was repeated 25 times to overcome slow and fast intensity fluctuations.

## Additional Information

**How to cite this article**: Kurpiers, J. and Neher, D. Dispersive Non-Geminate Recombination in an Amorphous Polymer:Fullerene Blend. *Sci. Rep.* **6**, 26832; doi: 10.1038/srep26832 (2016).

## Supplementary Material

Supplementary Information
