# Supplementary material for: Dispersive Non-Geminate Recombination in an Amorphous Polymer:Fullerene Blend
Source: Sci Rep. 2016 May 26;6:26832. doi: 10.1038/srep26832 (PMC4881019; doi:10.1038/srep26832)
Supplement: Supplementary Information [file srep26832-s1.pdf]

Supporting Information

Dispersive Non-Geminate Recombination in an Amorphous Polymer:Fullerene Blend

Jona Kurpiers and Dieter Neher*

Institute of Physics and Astronomy, Soft Matter Physics, University of Potsdam,

D-14476 Potsdam, Germany

Figures

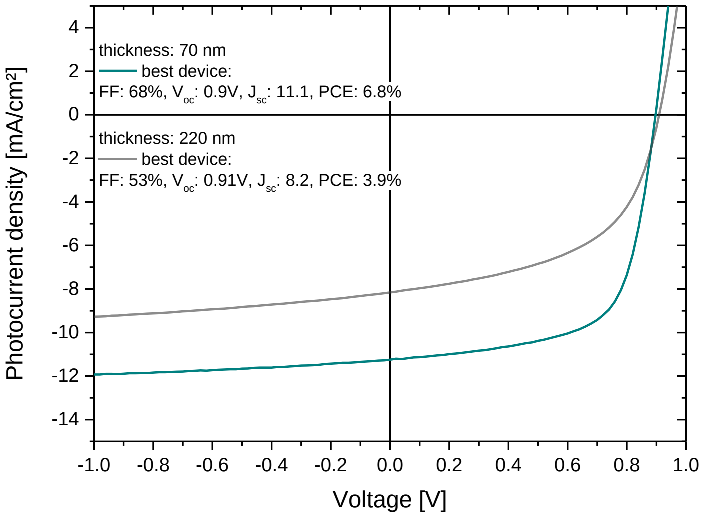

Figure SI1. Experimental JV-curves of 70 ^{o}C annealed optimized devices with an active layer thickness of 70 nm and 220 nm, as used in the TDCF experiments

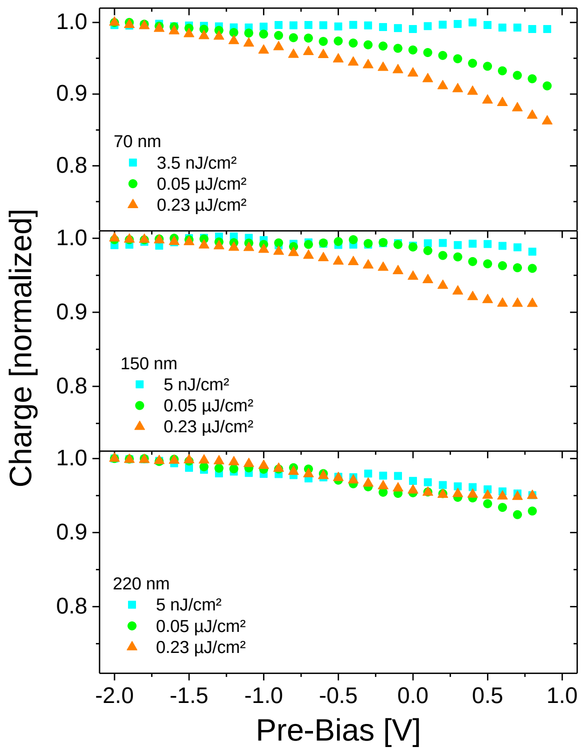

Figure SI2. Effect of layer thickness on the initial fast recombination loss. The total extracted charge normalized to -2V, measured for three different fluences, is plotted as a function of pre-bias for an active layer thicknesses of 70 nm, 150 nm and 220 nm. The decrease in extracted charge with increasing pre-bias becomes less fluence-dependence and overall weaker when the active layer thicknesses increases to 220 nm, indicating that the fast initial loss is far less significant in thicker active layers

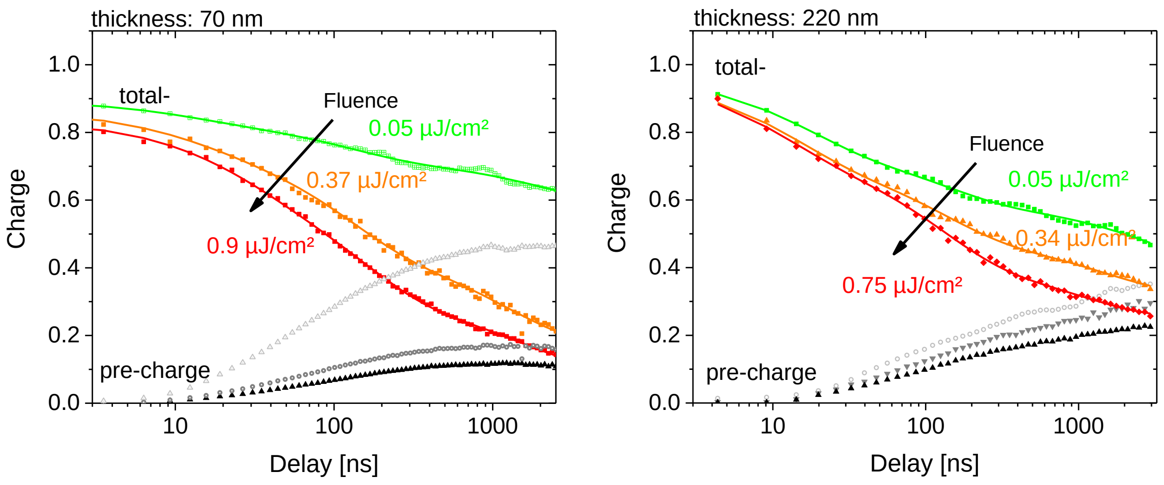

Figure SI3. TDCF transients for longer delay times: plotted is Q_{tot} and Q_{pre} (symbols) as a function of delay time for a 70 nm (left) and a 220 thick (right) active layer. Data have been normalized as in Figure Also shown are stretched exponential fits (solid lines), which were then used in the creation of the differential decay plots in Figure 4 for the thin 70 nm (left) and the 220 nm (right) device.

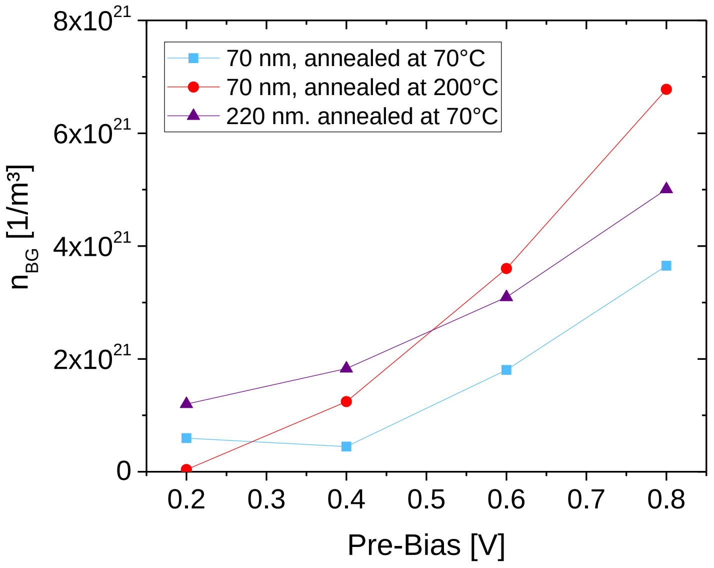

Figure SI4 Background charge as function of pre-bias in PCDTBT:PCBM: background charge was measured in the dark with bias assisted charge extraction (BACE) for the devices studied here. The continuous increase in the background charge density with increasing bias indicates that it origins mainly from injection.

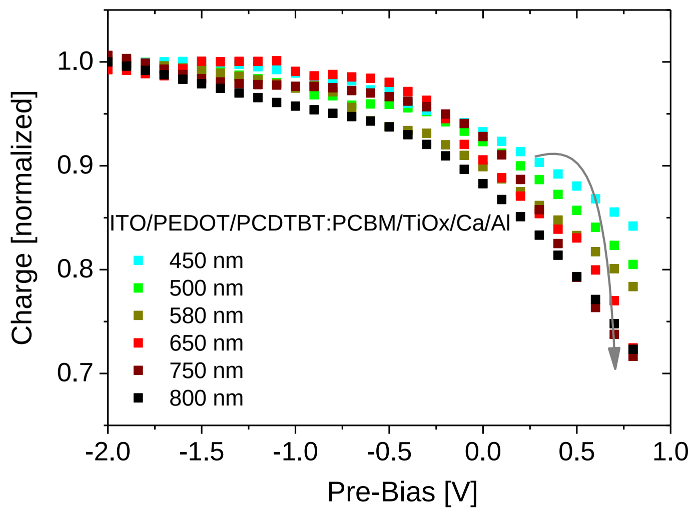

Figure SI5: Effect of excitation wavelength on the initial fast recombination loss. Shown is the normalized total extracted charge for a delay time of 12 ns. Excitation was with different excitation wavelengths, with the fluence adjusted to give approximately the same initial carrier density as for illumination at 532 nm with 0.1 µJ/cm². The active layer thickness was 70nm.

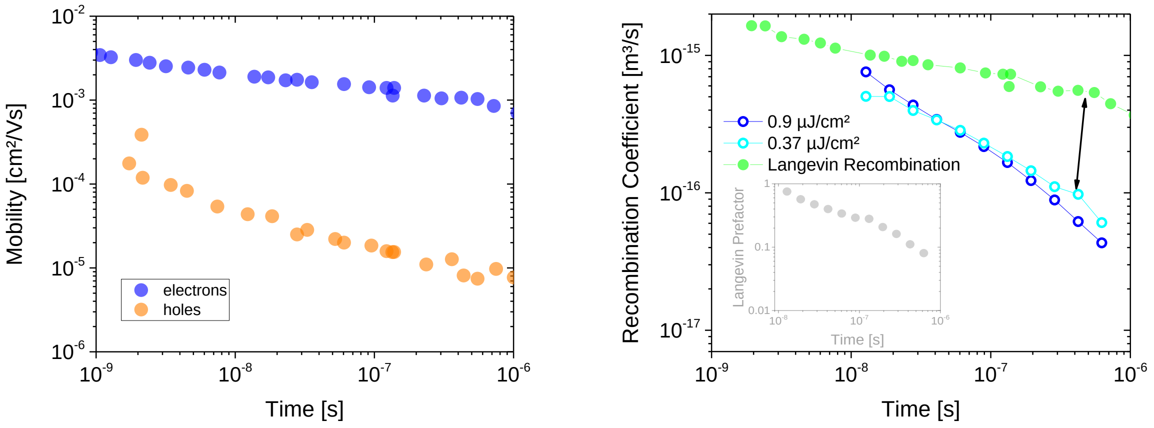

Figure SI6 Time dependent carrier mobility and recombination dynamics. Left: Time dependent electron and hole mobility in PCDTBT:PCBM taken from Figure S7 in Ref 1. Right: Comparison of the Langevin recombination coefficient (green filled circles) calculated from the time dependent mobilities with the time dependent bimolecular recombination coefficients obtained with TDCF experiments (from Figure 4f) for two different fluences. The Langevin pre-factor (Inset) derived from this comparison exhibits a continuous decrease as a function of time, from 0.7 at 10 ns to 0.08 at 1000 ns.

References

1 Melianas, A. *et al.* Photo-generated carriers lose energy during extraction from polymer-fullerene solar cells. *Nature Communications* **6**, 8778 (2015).
